# Effect of Pretreatment on Hydraulic Performance of the Integrated Membrane Process for Concentrating Nutrient in Biogas Digestate from Swine Manure

**DOI:** 10.3390/membranes10100249

**Published:** 2020-09-23

**Authors:** Yuanhang Zhan, Fubin Yin, Caide Yue, Jun Zhu, Zhiping Zhu, Mengyuan Zou, Hongmin Dong

**Affiliations:** 1 Institute of Environment and Sustainable Development in Agriculture, Chinese Academy of Agricultural Sciences, Beijing 100081, China; zzyh727@126.com (Y.Z.); carft_257@163.com (F.Y.); ycdhope@163.com (C.Y.); zhuzhiping@caas.cn (Z.Z.); condor1228@sina.cn (M.Z.); 2 Department of Biological and Agricultural Engineering, University of Arkansas, Fayetteville, AR 72701, USA; junzhu@uark.edu

**Keywords:** pretreatment, NF + RO, permeate, concentrate, biogas digestate

## Abstract

Nanofiltration (NF) or reverse osmosis (RO) process has been widely applied for concentrating nutrient in biogas digestate. However, efficient pretreatment is key to the sustainable operation of NF or RO. In this study, the combination of NF and RO for concentrating biogas digestate was compared using different pretreatments of hollow fiber ultrafiltration membrane (HFUFM) and ceramic membrane (CUFM). Pilot-scale batch tests were conducted (500 L). CUFM showed a higher membrane flux than HFUFM (100 ~ 180 L·(m^2^·h)^−1^ vs. 17 ~ 35 L·(m^2^·h)^−1^), but they showed little impact on the NF + RO process. Membrane fluxes of NF and RO were 20 ~ 48 L·(m^2^·h)^−1^ and 16 ~ 40 L·(m^2^·h)^−1^, respectively. In the RO permeates, the removal rates of total suspended solids (TSS), total solids (TS), chemical oxygen demand (COD), total nitrogen (TN), NH_4_^+^-N, and Cl^−^ were above 91%. In the concentrates, TN and total potassium (TK) were concentrated by 1.60 and 2.00 folds in the NF stage, and by 2.10 and 2.30 folds in the RO stage. Further attention should be paid to the antibiotics risks in the concentrates before they are utilized as plant fertilizers.

## 1. Introduction

Anaerobic digestion (AD) is a useful technology to treat swine manure [1], which generates biogas as a green energy but a large volume of digestate at the same time [2]. The digestate is rich in nitrogen, phosphorus, potassium, amino acid, and other biologically active substances [3,4,5], which can be utilized as organic fertilizer [6] to improve plant growth and quality [7]. However, the large volume of digestate could exceed the soil receiving capacity by irrigation [3,8], direct discharge to crop field will pose a high pollution risk to the environment due to runoff and seepage [4,6]. Thus, it is imperative to effectively treat the large amount of biogas digestate before discharging.

Membrane separation technology has been widely used in industrial processes [9,10,11,12,13]. It has also been employed as a new process for the high-quality processing of biogas digestate [8,14,15,16]. The membrane separation technologies used in biogas digestate treatment include microfiltration membrane (MF), ultrafiltration membrane (UF), nanofiltration membrane (NF), and reverse osmosis (RO). Among them, MF and UF are commonly used as pretreatment for NF and RO, a common practice for further concentration of nutrients [16,17]. Previous studies showed that clean permeates could be obtained for recycling by the NF and RO processes [8,18,19], and the concentrates with concentrated nutrients and reduced volume could be obtained as a valuable organic fertilizer [18,20,21,22]. Additionally, studies showed that the combined process of NF and RO (NF + RO) had lower energy consumption and better nutrient concentration performance than the single process, either NF or RO [23].

However, since the biogas digestate contained a large amount of suspended solids and organic matter [8,24,25,26], which could cause membrane blockage during the NF or RO concentration process [27,28,29], effective physical pretreatments of the digestates are required. The working efficiency of the NF or RO process could be significantly improved with membrane pretreatment [2,8,20]. The common fouling-resistant membrane materials for pretreatment include inorganic ceramic membrane (CUFM) and organic hollow fiber ultrafiltration membrane (HFUFM).

CUFM and HFUFM have been reported as pretreatment of biogas digestate or swine manure wastewater in many studies. Zacharof (2014) [30] tested a ceramic microfiltration membrane with a pore size of 200 nm to pretreat the biogas digestate from a swine farm, the results showed that the membrane system was capable of processing up to 140 L·(m^2^·h)^−1^ volume, reducing by 20.75% total solids and by 48.58% coarse particles. Pieters et al. (1999) [31] applied ceramic membrane with a pore size of 0.1 μm to treat swine farm wastewater, the mean membrane flux was 159 L·(m^2^·h)^−1^, the microfiltrate rejected 100% suspended solids and was fed to the RO system. Waeger et al. (2010) [32] pretreated the biogas digestate from a swine farm using ultrafiltration membrane with a pore size of 50 nm, the membrane permeation flux decreased from 40 to 25 L·(m^2^·h)^−1^, and a removal of 85% of the chemical oxygen demand (COD) was observed. Fugère et al. (2005) [33] used a hollow fiber ultrafiltration membrane with a pore size of 0.01 μm to pretreat swine farm wastewater, the suspended solids was removed by 100% and the coliforms were removed at an efficiency effectiveness greater than 99%. Zhan et al. (2018) [34] pretreated the biogas digestate from a swine farm with a hollow fiber ultrafiltration membrane with a pore size of 10 ~ 100 nm, the membrane flux was in the range of 9.0 ~ 16.7 L·(m^2^·h)^−1^, over 95% of suspended solids was removed in the ultrafiltration permeate. These studies all showed that both CUFM and HFUFM could be applied as a pretreatment method for removing suspended solids.

However, there is no direct comparison between the effects of HFUFM and CUFM pretreatments on the performance of the NF or RO concentration process. Therefore, this study compared the hydraulic performance and nutrient concentration of an integrated process of NF + RO with HFUFM and CUFM pretreatments, using the biogas digestate from swine manure as the substrate. The objective was to provide scientific information for selecting membrane-based pretreatment methods for biogas digestate concentration in applications.

## 2. Materials and Methods

### 2.1. Setup of Integrated Membrane Process

**Figure 1:**
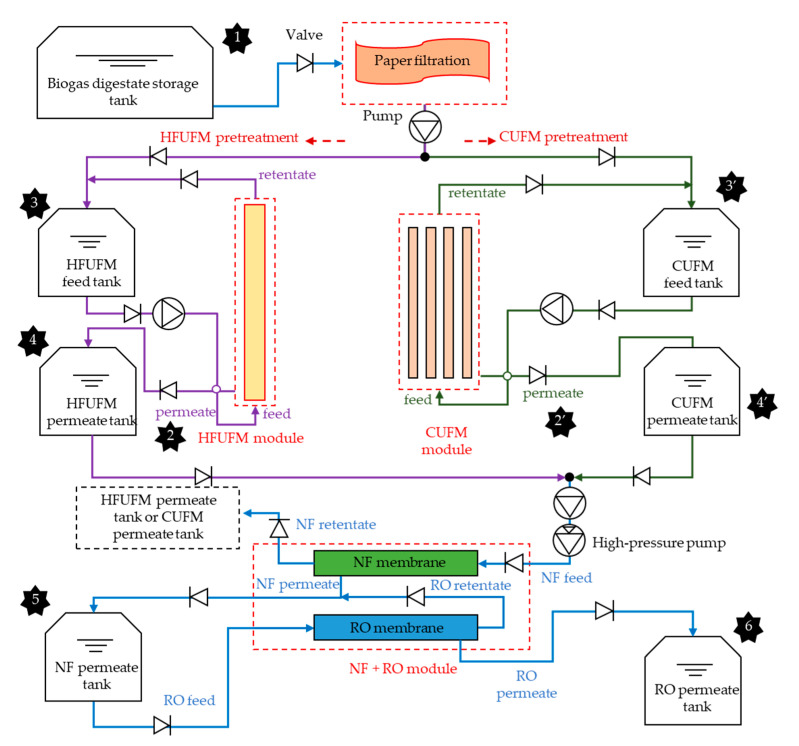
Schematic of the two combined processes under HFUFM and CUFM pretreatment. Sampling points 1 ~ 6: The original biogas digestate liquid (**1**), the HFUFM permeate (**2**), the CUFM permeate (**2′**), the HFUFM concentrate (**3**), the CUFM concentrate (**3′**), the NF concentrate (with HFUFM pretreatment (**4)**, with CUFM pretreatment (**4′**)), the RO concentrate (**5**), the RO permeate (**6**).

A pilot-scale integrated membrane system consisting of hollow fiber ultrafiltration membrane (HFUFM) and ceramic membrane (CUFM) pretreatment followed by a combined nanofiltration (NF) and reverse osmosis (RO) process (NF + RO) was built to concentrate biogas digestate (Figure 1). A tubular HFUFM module and a tubular CUFM module were used as separate pretreatments, the technical specifications of the two membrane modules were shown in Table 1. The two membrane modules chosen were the most commercially employed UF membrane modules. The membrane material and also the manufacturing method caused the difference in membrane pore size. The tubular NF and RO membrane were placed parallelly and horizontally, where a regular pump and a high-pressure pump were used to provide the pressure. The parts and materials for the membrane system including a paper filter, the CUFM module, the HFUFM module, and the NF and RO modules were supplied from the Hangzhou Rui Na Membrane Technology Co. Ltd. (Hangzhou, China). The technical specifications of NF and RO modules were shown in Table 1.

### 2.2. Experiment Design

The biogas digestate was collected from a digestate storage pond in a large-scale swine farm in Hebei Province, China. The biogas digestate was randomly drawn into a 1 m^3^ water tank by a pump placed at the center of the pond, about 1 m below the surface. Four batches of biogas digestate (1 m^3^ each) were collected on four different days (shown in Table S1) at the same sampling location in December 2017.

Four cross-repetition tests [10] were carried out for the treatment of the biogas digestate with a feed volume of 500 L, using the combined concentrating process of NF + RO coupled with either the HFUFM pretreatment or the CUFM pretreatment, i.e., HFUFM + NF + RO and CUFM + NF + RO. The two integrated processes with four replicates ran in the following random sequence, i.e., the first process underwent experimental batches of 1, 3, 6, and 8, and the second process covered experimental batches of 2, 4, 5, and 7 (Table S1).

The working conditions of the HFUFM, CUFM, NF, and RO membrane modules were maintained following the manuals or the information from the existing research [23], the crossflow velocity is 0.12 m·s^−1^ for HFUFM and 3.5 m·s^−1^ for CUFM; the applied pressure is 0.6 bar, 3 bar, 6 bar and 15 bar for HFUFM, CUFM, NF, and RO modules, respectively; the volume concentration ratio (VCR, the ratio of the concentrate volume to the feed volume) of HFUFM and CUFM were both 1/6 and that of NF and RO processes were both 1/5. A short period of clean water washing, was performed to achieve a better recovery of initial permeate flux after each treatment of the batch of biogas slurry. The flux, the inlet and outlet pressures of the membrane modules, the water supply pressure, and the liquid temperature were measured at 5 min intervals. The flux was measured using a floating flow meter and the volume was measured using a tank level sensor. A pressure gauge and a temperature sensor were used to record the liquid pressure and temperature. All sensors were supplied by the Hangzhou Rui Na Membrane Technology Co. Ltd. (Hangzhou, China). Samples from the inlet liquid, the HFUFM permeate, the CUFM permeate, the HFUFM concentrate, the CUFM concentrate, the NF concentrate, and the RO permeate and concentrate were collected (Figure 1).

### 2.3. Physico-Chemical Characterization of the Biogas Digestate from Swine Manure

The physico-chemical characteristics of the biogas digestate from the swine manure and the analysis methods used were presented in Table 2. The physico-chemical parameters analyzed included pH, turbidity, electrical conductivity (EC), total suspended solids (TSS), total solids (TS), volatile solids (VS), total phosphorus (TP), total nitrogen (TN), ammonia nitrogen (NH_4_^+^-N), chemical oxygen demand (COD), total organic carbon (TOC), metal ion (potassium, K^+^ or total potassium (TK); calcium, Ca^2+^; sodium, Na^+^; magnesium, Mg^2+^), anion (chloride, Cl^−^; carbonate, CO_3_^2−^; bicarbonate, HCO_3_^−^), and antibiotics (Sulfadimethylpyrimidine, enrofloxacin, ciprofloxacin, oxytetracycline, doxycycline).

### 2.4. Calculations and Statistical Analysis

In the study, the data were compiled, calculated, analyzed, and plotted using Microsoft Excel 2016 (Microsoft, Redmond, WA, USA). Analysis of variance was conducted based on the independent sample *t* test at a significance level of 0.05, using the statistical package of IBM SPSS Statistics 20.0 (IBM, Armonk, NY, USA).

The removal rate, Rr (%), denoted the percent reduction of the targeted substance in the permeate according to the following equation [35]:

(1) $$\mathrm{Rr}\left(\%\right)=\frac{C_{i}-C_{p}}{C_{i}}\times 100$$

where *C_i_* was the concentration or content of the substance in the separated liquid and *C_p_* was the concentration or content of the substance in the permeate.

The nutrient concentration factor, CF, was used to assess the concentration of the targeted substance in the concentrate according to the following equation [36]:

(2) $$\mathrm{CF}=\frac{C_{c}}{C_{i}}$$

where *C_c_* was the concentration or content of the substance in the concentrate and *C_i_* was the concentration or content of the substance in the initial feed of biogas digestate.

The volume concentration ratio (VCR) was defined as [8]:

(3) $$\mathrm{VCR}=\frac{V_{i}}{V_{i}-V_{p}}$$

where $V_{i}$ was the volume of the initial feed of biogas digestate, $V_{i}$ and $V_{p}$ were the volumes of the influent and the permeate of the tested membrane process.

The membrane flux decline rate (FD, %) was defined as [8]:

(4) $$\mathrm{FD}\left(\%\right)=\frac{J_{i}-J_{e}}{J_{i}}$$

where *J_i_* was the initial membrane flux (L·(m^2^·h)^−1^) in a batch process, and *J_e_* was the membrane flux (L·(m^2^·h)^−1^) at the end of a batch process.

## 3. Results and Discussion

### 3.1. Influence of HFUFM and CUFM on the Flux Changes

#### 3.1.1. Fluxes of HFUFM and CUFM Pretreatment

**Figure 2:**
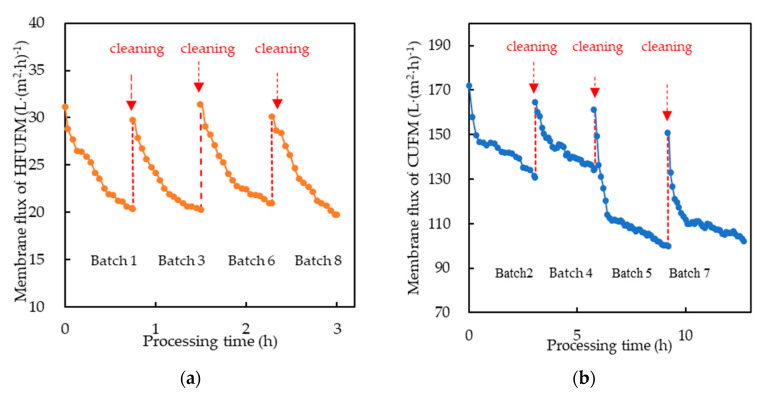

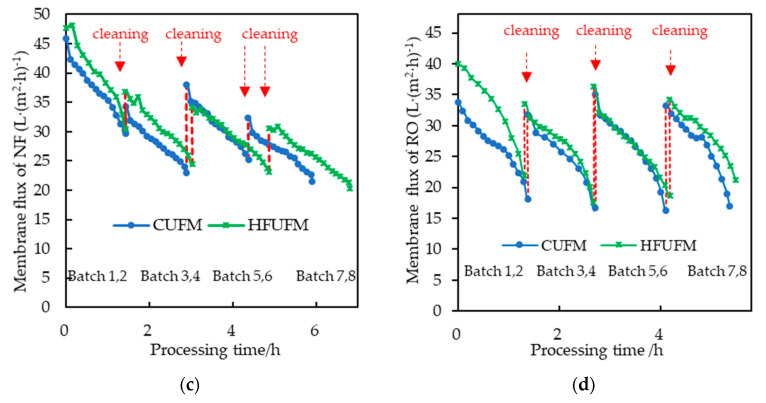
Membrane flux changes of (**a**) HFUFM pretreatment, (**b**) CUFM pretreatment, and (**c**) nanofiltration membrane (NF), (**d**) reverse osmosis (RO) process with HFUFM and CUFM pretreatment method.

The membrane fluxes of HFUFM and CUFM both showed a rapid decline with the running time (Figure 2a,b). The CUFM membrane permeation flux decreased from 180 to 100 L·(m^2^·h)^−1^, and the HFUFM membrane permeation flux decreased from 35 to 17 L·(m^2^·h)^−1^, with the former being about 5 times that of the latter, indicating that the membrane permeation property (membrane flux) of CUFM pretreatment was much higher. The average flux decline rate (FD, %) for CUFM and HFUFM was 37.08% ± 4.54% and 37.56% ± 5.28%, respectively, showing that there were no significant differences (*p* > 0.05), which indicated that the fouling characteristics of CUFM and HFUFM were similar in the batch process. The decline of the membrane flux was due to the forming of the cake layer on the membrane surface, which caused membrane fouling and resulted in a decrease of the membrane permeability [37]. The results were similar to the data from other studies presented in Table 3. The relatively high permeation property of the CUFM membrane was probably related to factors such as the pore size, membrane material, and applied pressures [10,30,32,38].

The larger working pressure of CUFM (3 bar) than that of HFUFM (0.6 bar) was one of the factors that contributed to a higher permeation property (membrane flux) of CUFM. In addition, CUFM had a larger pore size (200 nm) than HFUFM (10–100 nm), they could be reasons for CUFM to achieve a higher permeation property. CUFM, which was made of inorganic ceramic, had a relatively higher crossflow velocity (3.5 m·s^−1^, provided by the manufacturer) than HFUFM (0.12 m·s^−1^, provided by the manufacturer), which was made of organic polymers. The higher crossflow velocity contributed to the higher membrane flux according to Salud, et al. (2019) [38].

#### 3.1.2. Fluxes of Combined NF+RO Process

The variations of NF and RO membrane permeation fluxes with time for each experimental batch were shown in Figure 2c,d. The membrane permeation flux of NF varied from 20 to 48 L·(m^2^·h)^−1^ with the HFUFM pretreatment, and from 22 to 46 L·(m^2^·h)^−1^ with the CUFM pretreatment, respectively. The membrane flux of RO varied from 16 to 34 L·(m^2^·h)^−1^ with the HFUFM pretreatment, and from 18 to 40 L·(m^2^·h)^−1^ with the CUFM pretreatment, respectively. The slightly higher flux in NF and RO using HFUFM pretreatment than that using CUFM pretreatment was observed (Figure 2c,d). This little difference was probably due to the detected temperature difference of the inlet fluid from CUFM and HFUFM permeate. Studies [23,39] showed that a higher temperature of the inlet fluid of the NF and RO might cause the membrane flux to be higher. Therefore, compared to the CUFM, the higher temperature of HFUFM permeate might be the reason for a bit higher flux in NF and RO using HFUFM pretreatment. The average flux decline rate (FD, %) for NF process with CUFM and HFUFM pretreatment was 33.83% ± 1.55% and 33.9% ± 1.01%, respectively, and the average FD for RO process with CUFM and HFUFM pretreatment was 44.78% ± 4.18% and 48.98% ± 2.64%, respectively. The results showed that there were no significant differences (*p* > 0.05) between the two pretreatments in both cases. On the whole, the different pretreatments showed no noticeable effect on the fluxes of both the NF and RO processes due possibly to the same operating regime and feed volume used for the NF + RO process.

As many kinds of molecules and ions were rejected and accumulated on the surface of NF and RO membrane, a blocking layer could be formed [28,40,41,42], which reduced the permeability of the membranes. In addition, the concentrations of molecules and ions also increased in the influent, which could further reduce the membrane permeability, resulting in the rapid decline of membrane fluxes of both NF and RO process (Figure 2c,d). However, after each cleaning, the permeability of the membranes was largely re-established because the molecules and ions accumulated on the membrane surface were removed [20], which rejuvenated the membrane permeability, leading to the recovery of the membrane permeation fluxes.

### 3.2. Characteristics of CUFM Permeate and HFUFM Permeate

The physico-chemical properties of CUFM permeate and HFUFM permeate were shown in Table 4. Comparing with the biogas digestate, both CUFM permeate and HFUFM permeate showed an effective effect in removing turbidity and total suspended solids (TSS), with a removal rate (Rr) for turbidity of 95.50% ± 0.87% and 90.20% ± 1.04%, and a removal rate for TSS of 92.60% ± 1.67%, 89.36% ± 2.23%, respectively. The Rr of both turbidity and TSS in CUFM permeate was a little higher than that in HFUFM permeate, this might be due to the smaller membrane filtration area of CUFM (Table 1, 0.96 m^2^) than that of HFUFM (30 m^2^), which made the suspended solids have less chance to pass through and resulted in a relatively lower Rr. On the other hand, the Rr for COD was 29.13% ± 1.87% and 28.76% ± 1.10%, the Rr for TS was 50.00% ± 1.40% and 46.61% ± 3.04%, the Rr for TP was 60.65% ± 0.76% and 65.73% ± 0.93%, in CUFM and HFUFM permeate, respectively. The relatively high Rr for TP in both permeates might be related to the phosphorus solidification. Masse, et al. (2005) [24] reported that phosphorus was predominantly linked to particles between 0.45 and 10 mm. The Rr for EC, VS, TN, NH_4_^+^-N and antibiotics was below 20% both in CUFM permeate and HFUFM permeate, and the content of K^+^, Ca^2+^, Na^+^, Mg^2+^, Cl^−^, and HCO_3_^−^ didn’t decrease much compared with the content in the biogas digestate. These results were similar to other studies mentioned in the introduction part, which showed that UF pretreatment only had a significant effect on removing suspended solids, which would not only reduce membrane fouling of the NF and RO process, but also retain nutrients for NF and RO concentration.

### 3.3. Characteristics of RO Permeates of the Integrated Process

The average volume permeation ratio of the RO process in the four batch tests with HFUFM and CUFM pretreatments showed no significant difference (52.68% ± 0.99%, 51.79% ± 1.03%, respectively). The physico-chemical properties of the RO permeate were shown in Table 5, and the associated Rrs were included in Figure S1. There were no significant differences (*p* > 0.05) in the RO permeate between HFUFM and CUFM pretreatments according to the Rrs of turbidity, TSS, TS, VS, COD, TOC, EC, TN, NH_4_^+^-N, K^+^, Ca^2+^, Na^+^, Mg^2+^, TP, and Cl^−^.

In general, as shown in Figure S1a, the Rrs were above 97% for turbidity, TSS, TS, and VS; above 91% for EC, TN, and NH_4_^+^-N; above 98% for COD and TOC; above 91% for EC, TN, and NH_4_^+^-N; above 98% for TP; above 95% for K^+^, Ca^2+^, Na^+^, and Mg^2+^; and above 95% for Cl^−^. The contents of COD, TSS, NH_4_^+^-N, and TP were lower than the allowable values in the *National Discharge Standard of Pollutants for Livestock and Poultry Breeding* (GB 18596-2001) [43]. The contents of COD, TSS, and Cl^-^ were lower than the allowable values in the *National* *Standards for Irrigation Water Quality* (GB 5084-2005) [44].

In early studies, Caide (2018) [23] used a CUFM membrane as pretreatment of swine farm biogas digestate and a tubular NF and RO membrane for further treatment. In the final RO permeate, COD and NH_4_^+^-N were removed by 97.60% and 88.50%, respectively. Ruan et al. (2015) [20] used a HFUFM membrane to pretreat the swine farm biogas digestate, and a polyamide RO membrane for further treatment. In the RO permeate, the contents of COD and NH_4_^+^-N were both lower than 50 mg·L^−1^, and the Rrs were higher than 90%. Similar results were observed in this study. The solid, organic matter, and ions in the RO permeate could be almost completely removed using either the HFUFM or CUFM pretreatment. The RO permeates produced could be reused for house backwashing, boiler cooling, etc.

For antibiotics of sulfadimethylpyrimidine, enrofloxacin, ciprofloxacin, and doxycycline in the RO permeate, they were completely removed with removal rate of 100%, as shown in Figure S1b. However, in the RO permeates with CUFM and HFUFM pretreatment, the Rrs of oxytetracycline were 76.8% ± 16.9% and 59.7% ± 9.3% with 19.0 ± 13.5 ng·mL^−1^ and 33.1 ± 7.4 ng·mL^−1^ left, respectively. In the RO permeate, the remaining oxytetracycline could still pose a threat to the environment [45,46]. Further treatment is needed before disposal.

### 3.4. Characteristics of the Concentrates of the Integrated Process

**Figure 3:**
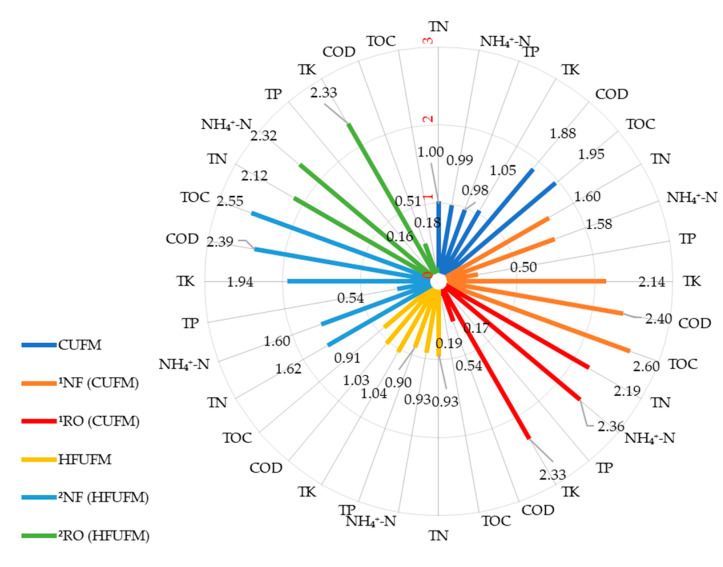
The average concentration factors of TN, NH_4_^+^-N, TP, TK, COD, and TOC in the concentrates of the integrated process with HFUFM and CUFM pretreatments. Note: ^1^NF (CUFM), ^1^RO (CUFM), and ^2^NF (HFUFM), ^2^RO (HFUFM) means NF concentrate, RO concentrate with CUFM pretreatment, and NF concentrate, RO concentrate with HFUFM pretreatment, respectively.

The volume concentration ratio (VCR) of HFUFM and CUFM pretreatments were both 6 and the VCR of NF and RO processes were both 5. The contents of TN, NH_4_^+^-N, TK, TP, COD, and TOC in different concentrates with HFUFM and CUFM pretreatments were shown in Figure S2. The nutrient concentration factors (CFs) were summarized as shown in Figure 3.

The contents of TN, NH_4_^+^-N, TK, and TP in the concentrates showed no significant difference between the HFUFM and CUFM pretreatments (Figure S2a–c), except that the TK content in the NF concentrate with the CUFM pretreatment was slightly higher than that with the HFUFM pretreatment (Figure S2d). The average CFs of TN and TK in the HFUFM and CUFM concentrates were close to 1, showing no concentration effect. But in the NF concentrates, the average CFs of TN, TK increased to 1.62 and 1.94 by HFUFM pretreatment, and 1.60 and 2.14 by CUFM pretreatment. In the RO concentrates, they increased to 2.12 and 2.33 by HFUFM pretreatment and 2.19 and 2.33 by CUFM pretreatment. The CFs of TP were close to or lower than 1 in the concentrates, indicating that there was no concentration effect for TP. This might be due to phosphorus solidification in the slurry [22].

In the HFUFM and CUFM concentrates, the CFs of COD were 1.03 and 1.88, respectively, and those for TOC were 0.91 and 1.95, respectively. The contents of COD and TOC in the CUFM concentrates were significantly higher than those in the HFUFM concentrates (*p* < 0.01), but they were not significantly different (*p* > 0.05) between the NF and RO concentrates (Figure S2e,f). COD and TOC were concentrated in the NF concentrates with HFUFM or CUFM pretreatment, the CFs were 2.40, 2.55 or 2.40, 2.60, respectively. But they were not concentrated in the RO concentrates. The CFs were blow 1, which indicated that COD and TOC were only rejected by the NF process. The contents of COD in the RO permeates with HFUFM and CUFM pretreatments were 380.00 ± 16.79 mg·L^−1^ and 402.00 ± 27.83 mg·L^−1^, which was close to the values in the discharge standard, GB 18596-2001 (400 mg·L^−1^).

The HFUFM and CUFM concentrates could be returned to the digestion process or the biogas digestate storage pond. The NF and RO concentrates both contained concentrated TN, NH_4_^+^-N, and TK, which was also reported in other studies [8,20,47]. Since N, P, K, and organic matter are the main plant nutrients [7,47], the concentrates produced can thus be used as a valuable organic fertilizer for crop growth. Moreover, the RO concentrates contained a higher concentration of N and K but a lower content of COD and TOC than the NF concentrates.

On the other hand, the NF and RO concentrates also contained antibiotics of sulfamethazine, enrofloxacin, ciprofloxacin, oxytetracycline, and doxycycline as shown in Figure S3. Thus, further attention should be paid to the antibiotics risks in the NF and RO concentrates before they could be utilized as an organic fertilizer for plants. In addition, the antibiotics content in the RO concentrates were significantly lower than that in the NF concentrates, indicating that the integrated NF + RO process could be a solution to reduce the risk of antibiotics in the RO concentrate.

## 4. Conclusions

HFUFM and CUFM pretreatments were applied for NF + RO process of treating biogas digestate (500 L), CUFM showed a higher membrane flux than that of HFUFM (100 ~ 180 L·(m^2^·h)^−1^ vs. 17 ~ 35 L·(m^2^·h)^−1^). NF + RO process performance with HFUFM and CUFM showed little difference. The flux of NF and RO decreased (20 ~ 48 L·(m^2^·h)^−1^ and 16 ~ 40 L·(m^2^·h)^−1^). In the RO permeates, TS, COD, TP, Cl^−^, EC, and TN were removed by above 91% and sulfadimethylpyrimidine, enrofloxacin, ciprofloxacin, and doxycycline were removed by 100%. In the concentrates (VCR = 5), TN and TK were concentrated about 1.60, 2.00 folds in NF stage, and about 2.10, 2.30 folds in RO stage. Further treatment for the antibiotics risks in the concentrates should be considered before they are utilized as plant fertilizers. Based on the results of effects on the membrane flux and the product quality, CUFM is suggested a better choice than HFUFM as the pretreatment of NF and RO in batch process. However, further studies should be conducted to investigate the fouling stability of the integrated membrane process in long run tests.

## Figures and Tables

**Table 1:** Characteristics of ceramic membrane (CUFM), organic hollow fiber ultrafiltration membrane (HFUFM), nanofiltration (NF), and reverse osmosis (RO) membrane modules.

Parameter	CUFM (Ceramic Membrane)	HFUFM (Hollow Fiber Ultrafiltration Membrane)	NF (Nanofiltration Membrane)	RO (Reverse Osmosis Membrane)
Material	Ceramic CRM 301940	Polyvinylidene fluoride (PVDF)	DOW NF 270-4040	DOW BW 30-4040
Filtration area (m^2^)	0.96	30	7.6	7.6
Pore feature (nm)	200	10–100	1–2	<1
Filtration mechanism	Sieving	Sieving	Selective permeability	Selective permeability
Max. pressure (bar)	4	3	30	30
Max. temperature (°C)	150	45	45	45
pH range	0–14	2–10	2–11	2–11

**Table 2:** The physico-chemical characteristics of swine manure digestate and the methods and equipment used for analysis.

Parameters	Analytical Method	Biogas Digestate Content
pH	pH glass electrode method	7.60 ± 0.04
Electrical conductivity (EC) (ms·cm^−1^)	Electrode method	7.44 ± 0.54
Turbidity (NTU)	Infrared scattering	318.8 ± 56.1
NH_4_^+^-N (mg·L^−1^)	Salicylic acid spectrophotometry	745.8 ± 12.3
Total nitrogen (TN) (mg·L^−1^)	Persulfate oxidation	662.5 ± 36.3
Chemical oxygen demand (COD) (mg·L^−1^)	Potassium dichromate rapid digestion spectrophotometry	588.5 ± 16.1
Total phosphorus (TP) (mg·L^−1^)	Ammonium molybdenum spectrophotometry	48.04 ± 3.77
Total suspended solids (TSS) (mg·L^−1^)	Filter paper drying weight method	134.2 ± 14.5
Total solids (TS) (mg·L^−1^)	Drying weight method	2263 ± 104
Volatile solids (VS) (mg·L^−1^)	Muffle furnace drying weight method	750.7 ± 112.7
Total organic carbon (TOC) (mg·L^−1^)	Combustion oxidation-non-dispersive infrared absorption method	210.8 ± 0.7
Total potassium (TK) (K^+^) (mg·L^−1^)	Inductively coupled plasma optical emission spectrometry	362
Ca^2+^ (mg·L^−1^)		32.6
Na^+^ (mg·L^−1^)		290
Mg^2+^ (mg·L^−1^)		46.6
Cl^−^ (mg·L^−1^)	Ion chromatography	304
HCO_3_^−^ (mg·L^−1^)	Acid–base indicator titration, potentiometric titration	3880
${CO}_{3}^{2-}$ (mg·L^−1^)		0
Sulfadimethylpyrimidine (ng·mL^−1^)	High performance liquid chromatography	405.8 ± 15.5
Enrofloxacin (ng·mL^−1^)		2.03 ± 0.03
Ciprofloxacin (ng·mL^−1^)		4.47 ± 0.02
Oxytetracycline (ng·mL^−1^)		82.12 ± 6.52
Doxycycline (ng·mL^−1^)		42.63 ± 4.10

**Table 3:** Studies applied HFUFM or CUFM process to pretreating the biogas digestate.

Applied Process	Pore Size (nm)	Working Pressure (Bar)	Membrane Flux Range (L·(m^2^·h)^−1^)	Compared with This Study	Studies
HFUFM pretreatment	50	0.3	40.0 ~ 25.0	slightly larger	[32]
	10 ~ 100	0.3	16.7 ~ 9.0	slightly lower	[34]
	10	1	40.0 ~ 25.0	similar	[33]
	10 ~ 100	0.6	35.0 ~ 17.0		This study
CUFM pretreatment	200	1	140.0 ~ 120.0	similar	[30]
	100	1.8	reached 159.0	similar	[31]
	200	3	80.0 ~ 120.0	similar	[23]
	200	3	180.0 ~ 100.0		This study

**Table 4:** The physico-chemical characteristics of the biogas digestate and the CUFM permeate and HFUFM permeate (mean ± SD).

Parameters	Biogas Digestate	CUFM Permeate	HFUFM Permeate
pH	7.60 ± 0.04	7.96 ± 0.02	7.90 ± 0.09
EC (ms·cm^−1^)	7.44 ± 0.54	6.61 ± 0.34	6.50 ± 0.63
Turbidity (NTU)	318.8 ± 56.1	15.90 ± 0.28	31.25 ± 0.38
NH_4_^+^-N (mg·L^−1^)	745.8 ± 12.3	528.50 ± 22.96	531.25 ± 27.27
TN (mg·L^−1^)	662.5 ± 36.3	562.50 ± 96.01	535 ± 78.26
COD (mg·L^−1^)	588.5 ± 16.1	546.00 ± 53.39	527 ± 48.42
TP (mg·L^−1^)	48.04 ± 3.77	18.90 ± 2.88	16.46 ± 2.92
TSS (mg·L^−1^)	134.2 ± 14.5	10.67 ± 3.77	13.67 ± 5.73
TS (mg·L^−1^)	2263 ± 104	1131.33 ± 145.72	1208.00 ± 73.97
VS (mg·L^−1^)	750.7 ± 112.7	533.33 ± 54.09	598.67 ± 44.58
TOC (mg·L^−1^)	210.8 ± 0.7	172.63 ± 0.97	145.973 ± 1.64
TK (K^+^) (mg·L^−1^)	362	404	314
Ca^2+^ (mg·L^−^^1^)	32.6	48.9	46.4
Na^+^ (mg·L^−^^1^)	290	283	236
Mg^2+^ (mg·L^−^^1^)	46.6	47.4	46.3
Cl^−^ (mg·L^−^^1^)	304	281	275
HCO_3_^−^ (mg·L^−1^)	3880	3650	3420
${CO}_{3}^{2-}$ (mg·L^−1^)	0	0	0
Sulfadimethylpyrimidine (ng·mL^−1^)	405.8 ± 15.5	282.14 ± 46.9	399.90 ± 7.87
Enrofloxacin (ng·mL^−1^)	2.03 ± 0.03	1.94±0.04	1.98 ± 0.06
Ciprofloxacin (ng·mL^−1^)	4.47 ± 0.02	4.40 ± 0.11	4.44 ± 0.01
Oxytetracycline (ng·mL^−1^)	82.12 ± 6.52	55.71 ± 8.65	83.36 ± 6.54
Doxycycline (ng·mL^−1^)	42.63 ± 4.10	26.09 ± 1.11	35.23 ± 1.74

**Table 5:** The physicochemical properties of the RO permeate from the biogas digestate treated using the NF + RO processes with HFUFM and CUFM (mean ± SD).

Parameters	RO Permeate with HFUFM Pretreatment Method	RO Permeate with CUFM Pretreatment Method	Discharge Standard of Pollutants for Livestock and Poultry Breeding (GB 18596-2001) [43]	Standards for Irrigation Water Quality (GB 5084-2005) [44]
pH	8.76 ± 0.40	9.06 ± 0.03	-	5.5~8.5
EC (ms·cm^−1^)	0.39 ± 0.08	0.40 ± 0.06	-	-
Turbidity (NTU)	0.40 ± 0.20	0.22 ± 0.02	-	-
COD (mg·L^−1^)	3.0 ± 2.1	8.0 ± 1.2	400	150
TN (mg·L^−1^)	45.8 ± 8.0	49.0 ± 12.8	-	-
NH_4_^+^-N (mg·L^−1^)	45.8 ± 6.8	47.6 ± 7.9	80	-
TP (mg·L^−1^)	0.20 ± 0.17	0.68 ± 0.10	8.0	-
TSS (mg·L^−1^)	0.0 ± 1.6	0.0 ± 1.6	200	80
TS (mg·L^−1^)	62.7 ± 15.4	65.3 ± 4.1	-	-
VS (mg·L^−1^)	10.0 ± 4.3	12.0 ± 3.3	-	-
TOC (mg·L^−1^)	1.3 ± 0.1	1.5 ± 0.2	-	-
HCO_3_^−^ (mg·L^−1^)	70.9	67.8 ± 3.4	-	-
${CO}_{3}^{2-}$ (mg·L^−1^)	72.7 ± 3.7	93.9 ± 4.7	-	-
K^+^ (mg·L^−1^)	13.6 ± 0.7	14.9 ± 0.8	-	-
Ca^2+^ (mg·L^−1^)	1.08 ± 0.06	1.58 ± 0.08	-	-
Na^+^ (mg·L^−1^)	10.8 ± 0.5	11.6 ± 0.2	-	-
Mg^2+^ (mg·L^−1^)	0.65 ± 0.04	0.77 ± 0.39	-	-
Cl^−^ (mg·L^−1^)	14.6 ± 0.7	16.3 ± 0.9	-	350
Sulfadimethylpyrimidine (ng·mL^−1^)	2.1 ± 0.4	1.9 ± 0.5	-	-
Enrofloxacin (ng·mL^−1^)	0	0	-	-
Ciprofloxacin (ng·mL^−1^)	0	0	-	-
Oxytetracycline (ng·mL^−1^)	33.1 ± 7.4	19.0 ± 13.5	-	-
Doxycycline (ng·mL^−1^)	0	0	-	-

## Supplementary Materials

The following are available online at https://www.mdpi.com/2077-0375/10/10/249/s1, Table S1: The different batches of the biogas digestate from swine manure, Figure S1: The Rrs (%) of (a) physical and chemical parameters and (b) antibiotics content in RO permeates with HFUFM and CUFM pretreatment method. Figure S2: The content of (a) TN, (b) NH_4_^+^-N, (c) TP, (d) TK, (e) COD, (f) TOC in the concentrates of the integrated process with HFUFM and CUFM pretreatments. Figure S3: The content of (a) sulfadimethylpyrimidine, (b) enrofloxacin and ciprofloxacin, (c) oxytetracycline, (d) doxycycline in the concentrates of the integrated process with HFUFM and CUFM pretreatment.
