# Supplementary material for: Effect of Pretreatment on Hydraulic Performance of the Integrated Membrane Process for Concentrating Nutrient in Biogas Digestate from Swine Manure
Source: Membranes (Basel). 2020 Sep 23;10(10):249. doi: 10.3390/membranes10100249 (PMC7597954; doi:10.3390/membranes10100249)
Supplement: Supplementary file 1 [file membranes-10-00249-s001.pdf]

*Supplementary materials*

# Effect of Pretreatment on Hydraulic Performance of the Integrated Membrane Process for Concentrating Nutrient in Biogas Digestate from Swine Manure

**Yuanhang Zhan [1,2], Fubin Yin [1], Caide Yue [1], Jun Zhu [2], Zhiping Zhu [1], Mengyuan Zou [1], Hongmin Dong [1,*]**

[1] Institute of Environment and Sustainable Development in Agriculture, Chinese Academy of Agricultural Sciences, Beijing 100081, China; zzyh727@126.com (Y.Z.); carft_257@163.com (F.Y.); ycdhope@163.com (C.Y.); zhuzhiping@caas.cn (Z. Z.); condor1228@sina.cn (M.Z.)

[2] Department of Biological and Agricultural Engineering, University of Arkansas, Fayetteville, AR 72701, USA; junzhu@uark.edu (J. Z.).

* Correspondence: donghongmin@caas.cn; Tel.: +86-108-210-9979

**Table S1.** The different batches of the biogas digestate from swine manure.

| Experiment batch | Sampling batch | Sampling time | Temperature (℃) |
|---|---|---|---|
| 1 | 1 | 2017/12/5 | 12 |
| 2 | | | |
| 3 | 2 | 2017/12/8 | 10.5 |
| 4 | | | |
| 5 | 3 | 2017/12/12 | 9 |
| 6 | | | |
| 7 | 4 | 2017/12/16 | 8 |
| 8 | | | |

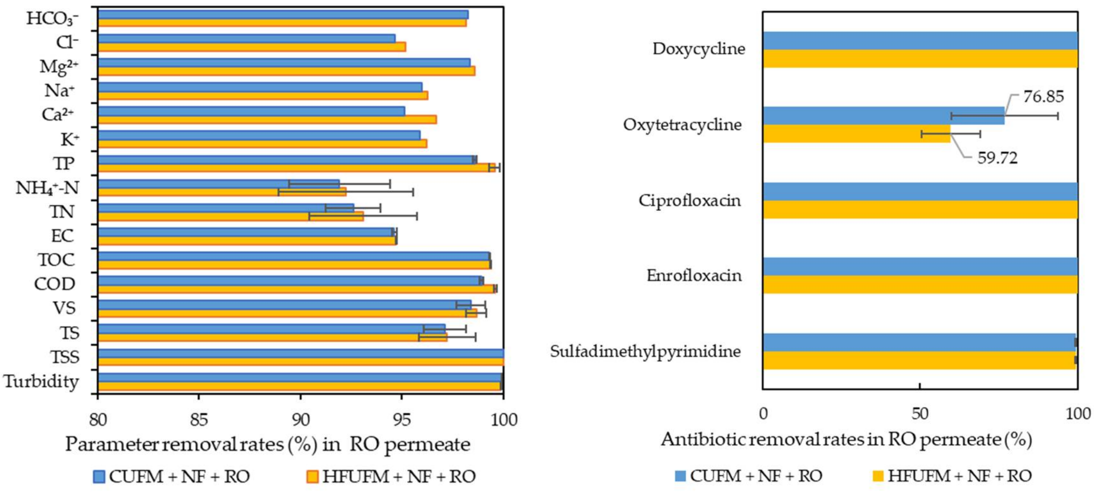


(**a**) (**b**)

**Figure S1.** The Rrs (%) of (**a**) physical and chemical parameters and (**b**) antibiotics content in RO permeates with HFUFM and CUFM pretreatment method.

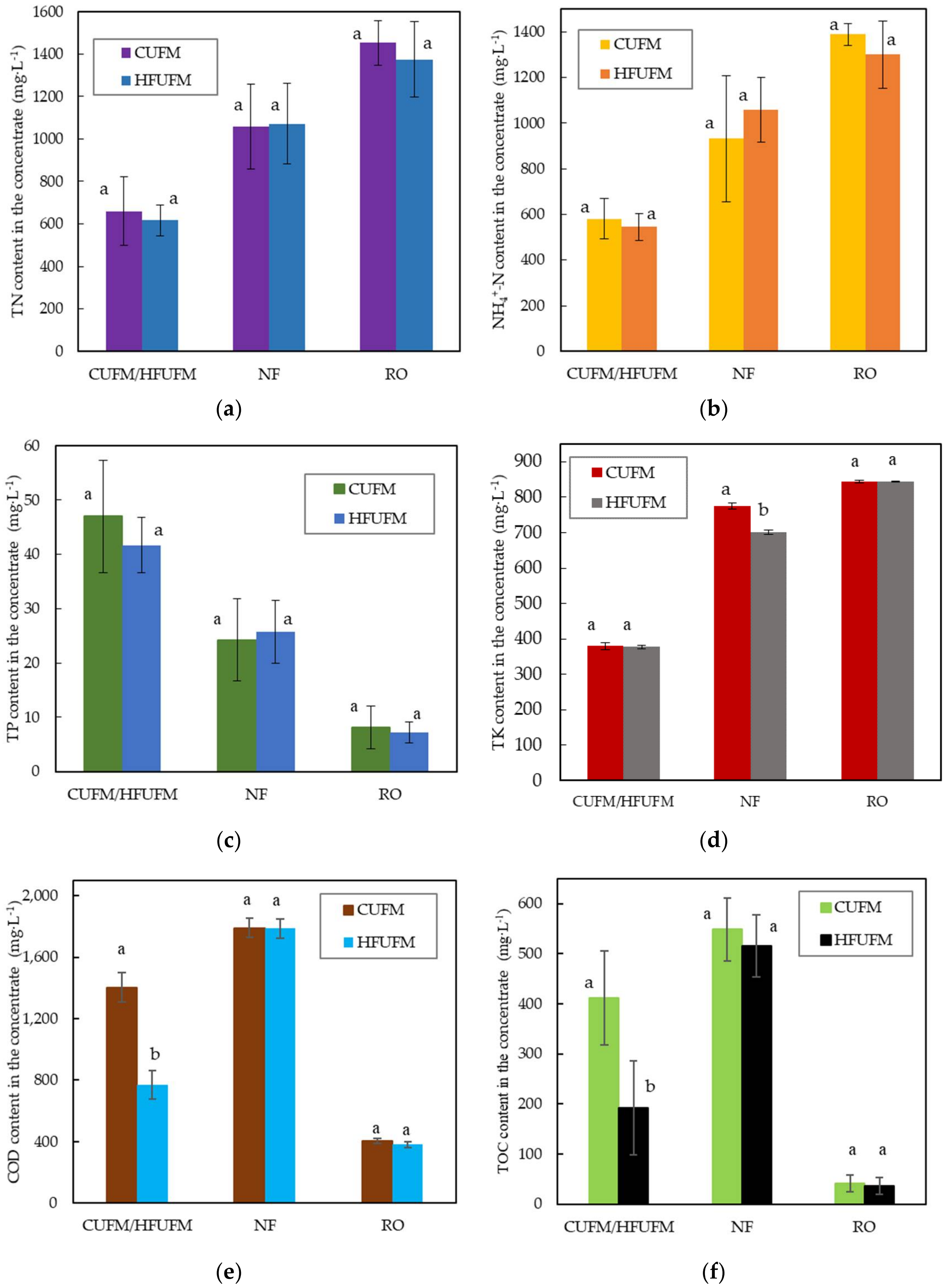


**Figure S2.** The content of (**a**) TN, (**b**) $NH_4^+$-N, (**c**) TP, (**d**) TK, (**e**) COD, (**f**) TOC in the concentrates of the integrated process with HFUFM and CUFM pretreatments.

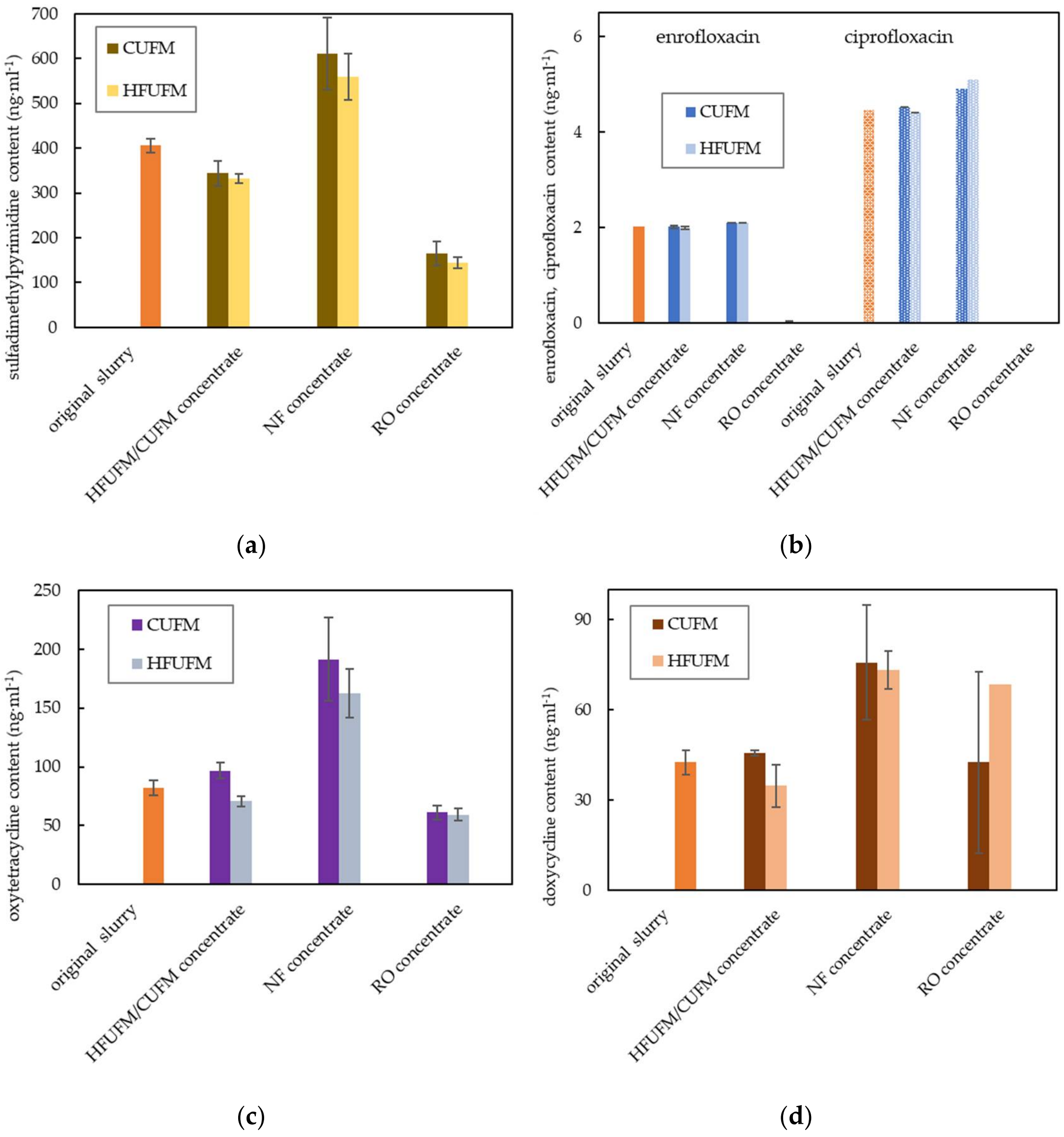


**Figure S3.** The content of (**a**) sulfadimethylpyrimidine, (**b**) enrofloxacin and ciprofloxacin, (**c**) oxytetracycline, (**d**) doxycycline in the concentrates of the integrated process with HFUFM and CUFM pretreatment.
